# Knowledge and use of wild edible plants in rural communities along Paraguay River, Pantanal, Brazil

**DOI:** 10.1186/s13002-015-0026-2

**Published:** 2015-05-30

**Authors:** Ieda Maria Bortolotto, Maria Christina de Mello Amorozo, Germano Guarim Neto, Jens Oldeland, Geraldo Alves Damasceno-Junior

**Affiliations:** Laboratory of Botany, Center of Biological Sciences and Health, Federal University of Mato Grosso do Sul (UFMS), C.P. 549, Campo Grande, Mato Grosso do Sul 79070-900 Brazil; Department of Ecology, IB, UNESP, C.P. 199, 13506-900 Rio Claro, São Paulo Brazil; Department of Botany and Ecology, Bioscience Institute, Federal University of Mato Grosso, 78 060-900 Cuiabá, Mato Grosso Brazil; Biodiversity, Evolution and Ecology of Plants, University of Hamburg, Ohnhorststrasse 18, 22609 Hamburg, Germany

**Keywords:** Traditional knowledge, Biodiversity, Ethnobotany, Wetland

## Abstract

**Background:**

Wild plants are used as food for human populations where people still depend on natural resources to survive. This study aimed at identifying wild plants and edible uses known in four rural communities of the Pantanal-Brazil, estimating the use value and understanding how distance to the urban areas, gender, age and number of different environments available in the vicinity can influence the knowledge and use of these plants by local people.

**Methods:**

Data on edible plants with known uses by communities were obtained through semi-structured interviews. A form with standardized information was used for all communities in order to obtain comparable data for analysis. For the quantitative analysis of the factors that could influence the number of species known by the population, a generalized linear model (GLM) was conducted using a negative binomial distribution as the data consisted of counts (number of citations).

**Results:**

A total of 54 wild species were identified with food uses, included in 44 genera and 30 families of angiosperms. Besides food use, the species are also known as medicine, bait, construction, technology and other. The species with the highest use value was *Acrocomia aculeata*. Older people, aged more than 60 years, and those living in more remote communities farther from cities know more wild edible plants. Statistical analysis showed no difference regarding gender or number of vegetation types available in the vicinity and the number of plants known by locals.

**Conclusion:**

This study indicated more knowledge retained in communities more distant from the urban area, indifference in distribution of knowledge between genders and the higher cultural competence of elderly people in respect to knowledge of wild edible botanicals.

## Background

Hundreds of wild plant species are used as food for human populations where people still depend on natural resources to survive. Ethnobotanical studies have been carried out to identify these species and their popular uses motivated by a strong interest in edible plants that are closely related to cultivated species [1] and which may offer greater global food security [2]. Furthermore, much of this knowledge is disappearing locally [3]; [4], making urgent to understand the factors responsible for this dynamics [5]. To understand the factors related to popular knowledge about wild edible plants, studies have sought information on the use value of species by comparing them to other categories of uses and linking them to the value of ecological importance [6], phytogeographic characteristics of the areas where they are located [7, 8] or proximity to the market town [9]. To find how socioeconomic factors influence the knowledge and use of edible wild plants, characteristics such as sex, age or the interaction of the two factors [10–17] were studied in rural areas.

Knowledge about food use is more associated to the most urban-distant remote communities [9] and also to those who have more diversity of vegetation types available [3, 7]. However, there is no pattern related to all of these variables in different communities, especially regarding gender difference in rural regions of the tropics. Sop et al. [11], for instance, observed no significant variation of knowledge of edible plants between genders in Burkina Faso. The same was observed in recent studies conducted in Brazil [12, 15]. However, results in rural regions of the tropics have shown the relationship between gender and knowledge and use of wild edible plants. Sometimes the men know more (10; 13, 17) and sometimes go down (18).

Concerning to age, studies have shown increased knowledge among the elderly [13, 14]. Phillips and Gentry [13] compared how knowledge on food use varies compared to other categories of uses, such as medicinal, e.g., in the community of Tambopata in the Peruvian Amazon. They observed that the knowledge of food use slowly increases with age and, apparently, most young adults and even children already know much of what is edible.

The analysis of factors related to knowledge in cultural, social and environmental contexts of communities is important, since it can be used to improve the life quality of the local population by bettering their diet and their income [18]. Understanding how distance to the urban areas, gender, age and number of different environments available can influence the knowledge and use of wild food plants by the local people would help in the construction of global strategies for biodiversity conservation [8] based on local knowledge [19].

Despite the considerable interest and development in this area, there is no ethnobotanical study discussing these factors in the Pantanal, Brazil. Wild edible plants have been used for many centuries, but there are few reports in the literature on food uses based on local knowledge. Indigenous populations were almost entirely decimated after the colonial period [20]. The Guató is presently the only indigenous community with traditionally occupied lands in Corumbá, Brazil [21].

The aim of this study was to investigate the knowledge and uses of wild edible plants in rural communities along Paraguay River, in the municipality of Corumbá, Brazil, and to examine the influence of gender, age, distance from communities to the nearest town and number of vegetation types (where communities are inserted) in communities’ knowledge. It is expected that greater knowledge will be found in older inhabitants; in the communities living far from the urban centers and in communities which have greater number of physiognomies available and between genders in knowledge about wild food plants.

## Material and methods

### Study area

**Fig. 1:**
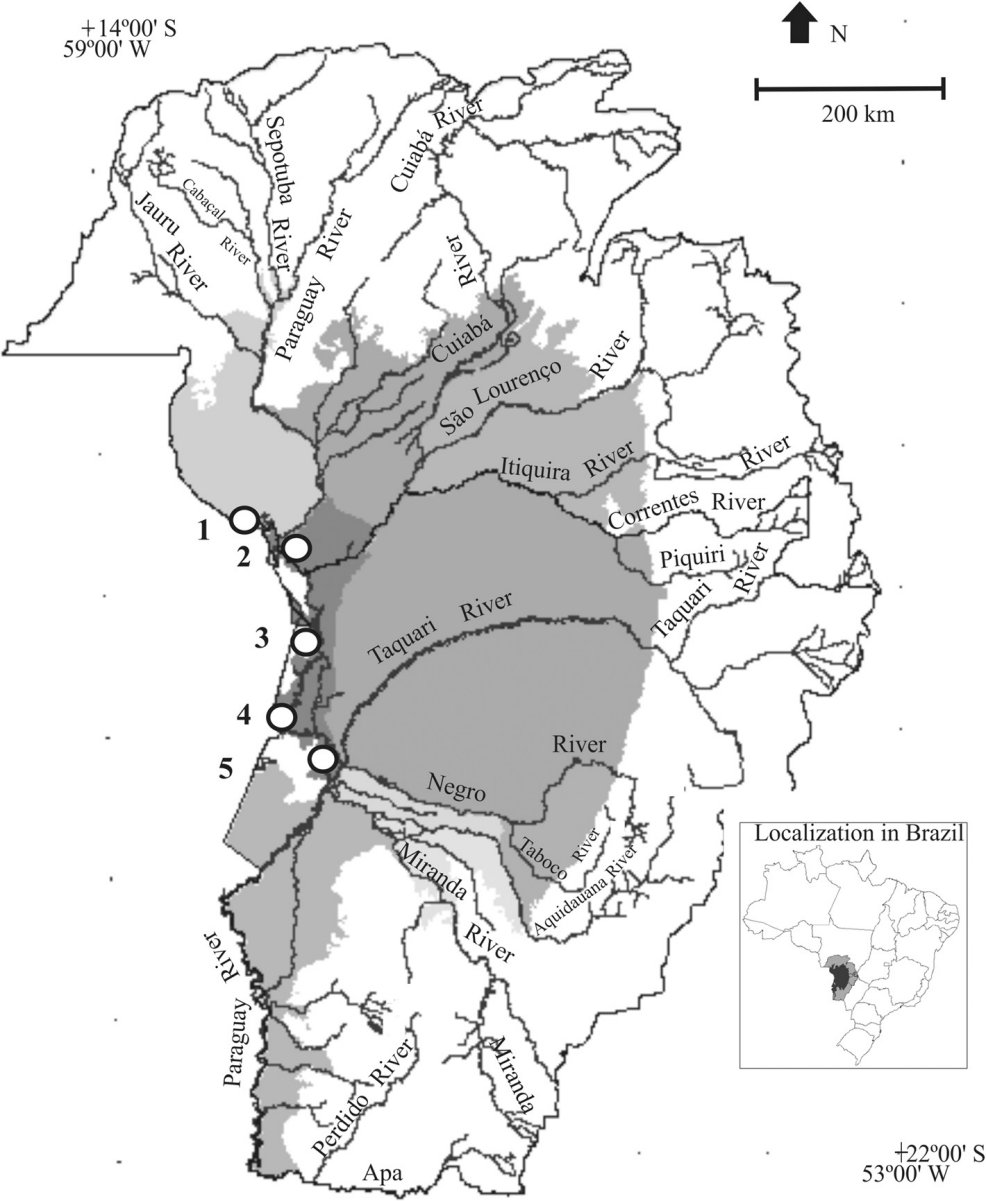
Brazilian Pantanal according to Silva & Abdon [63] showing the urban area of Corumbá (**4**) and the study communities along Paraguay River: Guató (**1**), Amolar (**2**), Castelo (**3**) and Albuquerque (**5**)

The Brazilian Pantanal is a vast floodplain, with about 140,000 km^2^, located in the center of South America [22] (Fig. 1). Along Paraguay River, which is an important waterway and the main collector of waters of the Pantanal, there are local communities that are among the oldest in the region [23]. These communities are settled in areas with access to various Pantanal vegetation types studied by [24], offering a good opportunity to investigate the knowledge of the local flora (Fig. 1).

The fieldwork was carried out in the rural communities of Albuquerque, Castelo and Guató (Table 1), located on the shores of the lakes Albuquerque, Castelo and Uberaba, respectively, and in the Amolar community located on the banks of the Paraguay River. The relief is characterized by extensive areas of floodplain and formations locally known as “morraria”, or “hills”, as they refer to areas of residual relief. These hills belong to residual reliefs of Urucum (with altitudes reaching 1065 m near the Albuquerque community) and Amolar (where the communities of Castelo, Amolar and Guató are located). The climate is tropical megathermal, with dry winters and rainy summers [25]. The flooding regime in the region is unimodal and fairly predictable, with a flood event per year, which has its peak between May and June.

**Table 1:** Basic information of the communities

Communities	Albuquerque	Castelo	Amolar	Guató
Latitude and Longitude (approx.)	19°S;57° W	18°S; 57° W	18°S; 57° W	17° S; 57° W
Occupation time (years)	228	+ − 120	+ − 130	+ − 10
Distance from Corumbá	60 km	94 km	198 Km	330 km
*Number of families*	+ − 300	+ − 30	9	16
Number of interviewees	29	25	9	8
Number of women (w) and men (m)	14 (w)	9 (w)	1 (w)	1 (w)
	15 (m)	16 (m)	8 (m)	7 (m)
Mean age	48	60	69	47
Age distribution: A. between 20 and 40 years; B (41–60); C (>60)	A (10)	A (5)	A (1)	A (3)
	B (13)	B (10)	B (3)	B (3)
	C (6)	C (10)	C (5)	C (2)
Number of vegetation types	5	3	5	3
Data collection	1997–1998	2001–2002	2001–2002	1997

Adapted from Bortolotto and Amorozo [18]

The vegetation is influenced by the flora of adjacent biomes such as the Amazon, Atlantic Forest, Cerrado and Chaco [26, 27]. The available vegetation types in surrounding communities includes floodplains [28–30], riparian forests on levees deposited by the Paraguay River along rivers and lakes [30], deciduous and semi-deciduous forests, and the Cerrado in the hills [24]. Surrounding communities of Castelo and Guató, Cerrado vegetation types and semidecidual forest are not observed and the vegetation cover in morraria is a deciduous forest [31] (Table 1).

### Studied communities

The communities of Albuquerque, Castelo, Amolar and Guató were formed at different times (Table 1). Albuquerque, Castelo and Amolar consist of residents who came from other Brazilian states or neighboring countries such as Bolivia, Argentina and Paraguay and indigenous people that lived in the area. The Guató inhabited in marshy and flooded plains of the upper Paraguay Basin and they lived predominantly in canoes [32]. Schmidt [33] reported that the Guató were on the verge of extinction in the beginning of the last century. They were considered extinct until the 1970s, when were located and recognized as Guató. Currently, they live in cities, isolated in rural areas, or reside on the island Ínsua [21] in traditionally occupied lands in Corumbá, Brazil. Among the islanders of Ínsua, there are families that have always lived there or in cities or farms of the Pantanal and now have chosen to live on the island. As a result of this life style, there are descendants of Guató among them constituting families of people with backgrounds similar to those of residents of other communities, creating a new generation with a greater ethnic mix.

The economy of these communities is based on agricultural production for subsistence, professional, artisanal and sport fishing, selling of bait to sport fishing and informal sale of manioc flour (*Manihot esculenta*). Environmental and economic changes spurred the migration of people to urban areas from the 1970s. The population is decreasing dramatically in the Castelo and Amolar communities, while the growth of the other two resumed from the 1990s onwards [23].

In the communities of Albuquerque, tourism has developed as an important activity from the 1980s [34] and in the Castelo community this has occurred since the late 1990s. In the communities of Amolar and Guató, there is a weak initiative in this sense. The Guató community in Insua Island shares socioeconomic characteristics with other communities along Paraguay River, such as the fact that they lived in urban areas or on farms in the region, and that they survive on activities such as fishing, livestock and small farm. The residents in Insua Island do not speak Guató language anymore, but Portuguese.

#### Data collection

Ethnobotanical data were collected through semi-structured interviews. Plants mentioned in the interviews were collected for identification. A form with standardized information was used for all communities in order to obtain comparable data for analysis, with items about age, gender and food use of plants as well as other purposes such as medicinal, bait, construction and others. The interviewees were selected based on their availability and on indications from other community members by using the snowball technique [35].

Seventy-one people, 25 women and 46 men were interviewed. The age ranged from 21 to 86 years, with average of 53.6 years. Women are mostly homemakers. Some also develop activities such as bait catching for fishing tourists, baker, midwife, farming, cooker and caretaker in tourists’houses (locally called “ranchos”). Regarding men, the main activities include farming, fishing, bait catching sold to fishermen and livestock. Often they develop more than one activity, such as catching baits and farm work, for instance.

The interviews on the island Ínsua were conducted in 1997 and data were collected to compose a study on the use of resources after the invitation from the Indian leader, in whose presence the interviews were conducted. Data collection in Albuquerque was developed during the period 1997–1998. Before starting the interviews, a meeting was organized in collaboration with the local school to explain the project objectives. In other communities, data collection was carried out in the period 2001–2002. Oral consent was obtained before interviews.

Data on the uses of plant species for various purposes was obtained. For the analysis, they were included in the categories of food, medicinal and bait use (eight or more species have been mentioned for each category) and other uses, with one to three species mentioned for each purpose. Thus, it was possible to estimate the number of mentions for food, medicinal or other purposes; which are the most used part; which are the most versatile plant for food and which ones have multiple uses (including in various categories of uses). Species used only in the interviewee’s childhood are included among the results stating that information.

Plants mentioned in the interviews were collected and deposited in the Herbarium of Federal University of Mato Grosso do Sul, *campus* of Corumbá (COR). In all communities, the respondents participated in the collection. In this paper, the term “wild” refers to species from native vegetation types of Pantanal [36].

#### Data analysis

For qualitative analysis, we considered the entire information of the respondents on food uses of wild plants, the parts used, popular names used by human populations in the past and present. Information about the use or abandonment of use mentioned by respondents was compared with the available literature. We considered only edible plants mentioned collected in natural vegetation. Cultivated species from other continents or vegetation types of South America mentioned in the interviews were not considered.

For the quantitative analysis of the factors that could influence the number of species known by the population, a generalized linear model (GLM) was conducted using a negative binomial distribution as the data consisted of counts (number of citations). Species richness per respondent was used as the dependent variable while respondent’s age and sex, number of vegetation types, and distance to the city served as independent variables. GLMs were calculated by using the free statistical programming language R [37].

The respondents’ age was divided into three categories: A. between 20 and 40 years (19 respondents); B. between 41 and 60 years (29 respondents); and C. over 60 years (23 respondents). The distance from the city was divided into two categories: up to 100 km and 200 km or more. Five vegetation types were considered: 1. floodplains (including aquatic plants); 2. riparian forests; 3. semideciduous forest; 4. deciduous forest; and 5. Cerrado, identified based on studies by [24, 26, 38, 39] and field observations by the team. We found three different vegetation types in the communities of Castle and Guató (floodplains, riparian forests and deciduous forest), and five in the communities of Amolar and Albuquerque. These variables were first analyzed using a GLM and non-significant variables were dropped in a stepwise manner. Only the significant variables were maintained and a final GLM was calculated.

Two use values for each species were calculated. The general use value was estimated using all the mentions for various uses obtained for each species cited as edible at least once. The use value considering only the number of mentions for food use was also estimated. The formula used was adapted from Philips and Gentry [13] by considering a single informant interview, as used by Ferraz et al. [40]: UVs = ΣUs/n; where: UVs = use value of species “s”; *n* = total number of respondents in the sample (*n* = 71); Us = number of citations of use mentioned by each informant for the species “s”. These use values were calculated two times: for all uses (general) and only for food use. Both use values were listed for each species in a table for interpretation.

## Results

### General aspects of plant uses and use value

We identified 54 wild species in 41 genera and 31 families of angiosperms (Table 2) with known uses as food by the residents of all four communities. The species with the higher use value (UV) was *Acrocomia aculeata* with UV below 1 (Table 2). Twenty species were mentioned exclusively for food use. Other species were also mentioned for medicinal purposes (16), bait (8), and others such as hay (3) technology (2) construction (2) bewitchment (2), ornamental (1), bee nesting (1) and firewood (1). The term “bewitchment” is used locally to refer to a ritual practiced to cure people’s diseases. The selling of fruits, seeds and other natural products was not mentioned, but products such as compote and vinegar with fruit *Plinia cauliflora* and liqueurs with *Caryocar brasiliense* and *Hancornia speciosa* were sometimes indicated.

**Table 2:** Wild edible species cited in the communities

Family	Species	Local name	Vegetation type	Edible plant parts	Mode of consumption	Other uses	UV (General)	UV (edible)
Anacardiaceae	*Spondias mombin* L.	Cajá, acaiá, caiá	Sd, Rf	Fruit	Ripe fruits eaten fresh	Bait	0.085	0.056
Annonaceae	*Annona cornifolia* A. St. – Hil.	Ata-do-campo	Ce	Fruit	Pulp ripe fruits eaten fresh	-	0.014	0.014
Annonaceae	*Annona nutans* (R.E.Fr.) R.E.Fr.	Ata-brava	An, De	Fruit	Pulp ripe fruits eaten fresh	-	0.014	0.014
Annonaceae	*Annona* sp.	Ata-do-campo	Ce	Fruit	Pulp ripe fruits eaten fresh	-	0.028	0.028
Apocynaceae	*Hancornia speciosa* Gomez	mangaba	Ce	Fruit	Pulp ripe fruits eaten fresh	Medicinal, forage, technology	0.141	0.099
Arecaceae	*Acrocomia aculeata* (Jacq.) Lodd. Ex Mart.	Bocaiuva, “Maguedji” (Guató)	An, De, Sd, Ce, Rf	Fruit (epicarp, mesocarp), seeds, leaf	Ripe fruit pulp (mesocarp) eaten fresh, liquor produced with pulp, sugar and alcohol, juice made with water and pulp after roasted in oven^a^; alcoholic beverage, fermented juice^a^, oil produced from the crushed seeds and placed in boiling water^a^, drink prepared with pulp and water, drink prepared with hot milk put over the grated seeds, palm heart baked^a^	Medicinal, bait, others (forage)	0.662	0.352
Arecaceae	Allagoptera leucocalyx (Drude) Kuntze	Buri	An, Ce	Fruit	Mesocarp and seeds consumed fresh	Forage	0.028	0.014
Arecaceae	*Attalea phalerata* Mart. ex Spreng.	Acuri, “mudjí ” (Guató)	An, De, Sd, Ce, Rf	Fruit, leaf	Pulp unripe or ripe fruits eaten cooked, palm heat baked^a^,	Construction, Technology, bait	0.197	0.085
Arecaceae	*Bactris glauscescens* Drude	Tucum-roxo, tucum-verde, “magueto” (Guató)	Rf	Fruit	Pulp ripe fruits eaten fresh	Bait, construction	0.366	0.028
Arecaceae	*Bactris major* Jacq.	Tucum-branco	Rf	Fruit	Pulp ripe fruits eaten fresh	Bait	0.042	0.028
Arecaceae	*Bactris riparia* Mart.	Tucum-vermelho	Rf	Fruit	Pulp ripe fruits eaten fresh	Bait	0.070	0.014
Arecaceae	*Copernicia alba* Morong	Carandá, “mufá” (Guató)	Sd, Rf, Fl	Fruit	Pulp ripe fruits eaten fresh	Baits, construction, others (handicraft)	0.310	0.028
Arecaceae	*Desmoncus orthacanthos* Mart.	Urubamba, tucum-preto	Rf	Fruit	Pulp ripe fruits eaten fresh	Bait, technology	0.042	0.014
Bromeliaceae	*Ananas ananassoides* (Baker) L.B.Sm.	Abacaxizinho	Ce	Fruit	Pulp ripe fruits eaten fresh	-	0.014	0.014
Cactaceae	*Cereus bicolor* Rizzini & A. Mattos	Urumbeva	De, Sd	Fruit	Pulp ripe fruits eaten fresh	Others (nests of bees)	0.028	0.014
Caryocaraceae	*Caryocar brasiliense* Cambess.	Pequi	An, Ce	Fruit	Pulp ripe fruits eaten cooked, liquor produced with pulp, sugar and alcohol	-	0.099	0.070
Cecropiaceae	*Cecropia pachystachya* Trécul	Embaúba	An, De, Sd, Ce, Rf	Fruit	Pulp ripe fruits eaten fresh	medicinal	0.225	0.014
Celastraceae	*Salacia elliptica* (Mart. ex Schult.) G. Don	Siputá, “mats´í” (Guató)	Sd, Rf	Fruit	Ripe fruits eaten fresh	Medicinal, technology	0.155	0.042
Chrysobalanaceae	*Couepia uiti* (Mart. & Zucc.) Benth. ex Hook.f.	Fruta-de-pato	Ce, Rf	Fruit	Pulp ripe fruits eaten fresh	-	0.028	0.028
Clusiaceae	*Garcinia gardneriana* (Planch. & Triana) Zappi	Acupari	Sd, Rf	Fruit	Pulp ripe fruits eaten fresh	Bait	0.070	0.028
Combretaceae	*Buchenavia tomentosa* Eichler	Tarumarana	Rf	Fruit	Pulp ripe fruits eaten fresh	Ornamental, others (forage)	0.056	0.014
Ebenaceae	*Diospyros hispida* A.DC.	Olho-de-boi	Ce	Fruit	Pulp ripe fruits eaten fresh	-	0.014	0.014
Fabaceae	*Cassia grandis* L.f.	Canafístula	Rf	Fruit	Resin of fruits (ripe)	Medicinal	0.056	0.014
Fabaceae	*Hymenaea courbaril* L.	Jatobá-mirim, jatobá-preto	De, Sd, Rf	Seeds (sarcotesta)	Farinaceous pulp (sarcotesta) eaten fresh	Medicinal	0.366	0.014
Fabaceae	*Hymenaea stigonocarpa* Mart. ex. Hayne	Jatobá-cascudo	Ce	Seeds (sarcotesta)	Sarcotesta eaten fresh	Medicinal	0.239	0.014
Fabaceae	*Inga vera* Willd.	Ingá	Rf	Fruit	Pulp (aryl seed) consumed fresh	Bait, construction, others (firewood for charcoal production)	0.099	0.028
Fabaceae	*Senna occidentalis* (L.) Link	Fedegoso	An, De, Sd, Ce, Rf, Fl	Seeds	Drink (café de fededoso) made with roasted and ground seeds	medicinal	0.211	0.014
Indeterminada	Indet.	Azedinha	Sd	Fruit	Drink produced with pulp	-	0.014	0.014
Lamiaceae	*Vitex cymosa* Bertero ex Spreng.	Tarumã, “madô”(Guató)	Sd, Rf	Fruit, flower	Ripe fruits eaten fresh and tea prepared with the flowers	Medicinail, bait	0.169	0.085
Malpighiaceae	*Byrsonima cydoniifolia* A.Juss Juss.	Canjiquinha	Rf, Fl	Fruit	Pulp ripe fruits eaten fresh	Bait	0.028	0.028
Malpighiaceae	Indet.	Cereja	Rf	Fruit	Ripe fruits eaten fresh	-	0.014	0.014
Malvaceae	*Guazuma ulmifolia* Lam.	chico-magro	An, De, Sd, Ce, Rf	Fruit	Fresh fruits	Medicinal	0.056	0.014
Malvaceae	*Sterculia striata* A.St.-Hil. & Naudin	Manduvi	De, Sd	Seeds	Seeds consumed toast	Others (sympathy to heal and medicinal - veterinary uses)	0.056	0.042
Melastomataceae	*Mouriri guianensis* Aubl.	Roncador	Rf	Fruit	Ripe fruits eaten fresh.	Forage	0.113	0.014
Menispermaceae	*Abuta grandifolia* Mart. Sandwith	Grão-de-galo	Sd	Fruit	Pulp ripe fruits eaten fresh	-	0.014	0.014
Moraceae	*Maclura tinctoria* (L.) D. Don ex Steud.	Taiúva	De, Sd	Fruit	Pulp ripe fruits eaten fresh	Medicinal	0.028	0.014
Myrtaceae	*Eugenia uniflora* L.	Pitanga	An	Fruit	Pulp ripe fruits eaten fresh	Medicinal	0.014	0.014
Myrtaceae	Indet.	Orvalho	De, Sd	Fruit	Ripe fruits eaten fresh.	-	0.014	0.014
Myrtaceae	*Plinia cauliflora* (Mart.) Kausel	Jabuticaba, jabuticaba-nativa	An, De	Fruit	Ripe fruits eaten fresh, like a jam and vinegar	Medicinal	0.352	0.324
Myrtaceae	*Psidium* sp.	goiabinha, goiabinha-do-mato	Sd	Fruit	Ripe fruits eaten fresh	-	0.028	0.014
Nynphaeaceae	*Victoria amazonica* (Poepp.) J.C. Sowerby	Forno d-água	Aq	Seeds	Starch made with seed, peeled and crushed in pestle^a^	-	0.014	0.014
Passifloraceae	*Passiflora cincinnata* Mast.	Maracujá-do-mato	De	Seeds	Pulp (seed aryl) consumed fresh	-	0.056	0.042
Passifloraceae	*Passiflora misera* Kunth	Maracujá-do-mato, maracujá-nativo	Rf	Seeds	Aryl seed consumed fresh	-	0.028	0.028
Poaceae	*Oryza glumaepatula* Steud.	Arroz, “matchamo” (Guató)	Aq	Seeds	Sun-dried seeds, peeled, and cooked^a^	-	0.014	0.014
Poaceae	*Oryza latifolia* Desv.	Arroz, “matchamo”(Guató)	Aq	Seeds	Sun-dried seeds, peeled, and cooked^a^	-	0.014	0.014
Polygonaceae	*Coccoloba parimensis* Benth.	Canjiquinha, rosarinho, uvinha	De, Rf	Fruit	Pulp ripe fruits eaten fresh	-	0.042	0.042
Rhamnaceae	*Rhamnidium elaeocarpum* Reissek	Cabriteira	De, Sd, Rf	Fruit	Ripe fruits eaten fresh	Medicinal, technology	0.056	0.014
Rhamnaceae	*Zizyphus oblongifolius* Moore.	Fruto-de-cabra, veludinho, “macariguá” (Guató)	De	Fruit	Ripe fruits eaten fresh	Others (oral hygiene)	0.056	0.028
Rubiaceae	*Alibertia edulis* (Rich.) A. Rich. ex DC.	Marmelada	Ce	Fruit	Pulp ripe fruits eaten fresh	Baits	0.028	0.014
Rubiaceae	*Genipa americana* L.	Jenipapo, “mató” (Guató)	An, Rf	Fruit	Mature pulp used to make jams and liquor	Medicinal, bait, technology, others (sympathy to heal)	0.254	0.070
Rutaceae	*Esenbeckia almawillia* Kaastra	Côca	Rf	Leaf	Leaves used to add on the hot “garapa” (sugar cane juice) and for making tea enjoyed by taste.	Medicinal	0.042	0.014
Sapindaceae	*Mellicoccus lepidopetalus* Radlk.	Água-pomba, “mapô” (Guató)	De	Fruit	Ripe fruits eaten fresh	-	0.099	0.099
Sapindaceae	*Talisia esculenta* (A. St.-Hil.) Radlk.	Pitomba	De, Sd	Fruit	Ripe fruits eaten fresh	Medicinal (veterinary uses)	0.070	0.042
Sapotaceae	*Pouteria glomerata* (Miq.) Radlk.	Laranjinha-de-pacu, “macondjê”(Guató)	Rf	Fruit	Ripe fruits eaten fresh	Bait	0.296	0.056

*De* deciduous forest, *Sd* semideciduous forest, *Ce* Cerrado, *Rf* riparian forest, *Fl* floodplains: An = anthropogenic (Homegardens and terrenos)

^a^Indicates only uses made in the past

Guató name “…” were compiled from Oliveira (1996)

Out of the 71 respondents, 18 did not mention wild edible plants. Among respondents of the two most distant communities, only one did not mention native edible plants, while in the two communities closest to the urban area, 17 respondents did not mention any wild edible plant. The Amolar community mentioned the highest number of species (29), followed by Albuquerque (24) Castelo (22) and Guató (10). The average of wild species mentioned as edible per resident in each community was 0.8 (Albuquerque), 0.9 (Castelo), 1.3 (Guató) and 3.2 (Amolar).

### Richness x age, distance of urban areas, gender and number of physiognomies

Elderly people more than 60 years old and those living in more remote communities, which were far from cities, knew more about wild edible plants (Table 3). The generalized linear model explained 22 % of variance, i.e., pseudo R^2^ = 0.22. Statistical analysis showed no difference in relation to gender, number of available vegetation types and the number of plants known by locals. Gender has not appeared in the table because it was eliminated in the stepwise regression process.

**Table 3:** GLM showing relation between number of species known and analyzed data

(Intercept)	Estimate	Std. Error	*z* value	Pr (>|z|)
	−0.683056	0.515851	−1.324	0.1855
Age	0.012229	0.005645	2.166	0.0303*
Number of vegetation types	0.161619	0.08931	1.81	0.0704 .
Distance from city	0.002931	0.001326	2.21	0.0271*

Signif. codes: *0.05

The most important vegetation types for wild edible resources obtainment were riparian (28 species), followed by semideciduous forest (20), deciduous forest (18), and Cerrado (16) species. Only three aquatic species were known by one indigenous community elderly person: *Oryza latifolia*, *Oryza glumaepatula* and *Victoria amazonica*.

Twenty five species occured in more than one vegetation type, and ten occurred also in anthropic areas that were remnants of native vegetation, along with species maintained by residents, such as in vacant lots and backyards. One resident mentioned that she planted two species from the Cerrado (*Hancornia speciosa* and *Caryocar brasiliense*) in the backyard because it was a fruit tree of her choice that could otherwise only be collected at more distant locations, such as on the slope, with access difficulties to it due to her age.

These species mentioned as edible and collected species in riparian forests are also mostly used as fishing bait, and include most Arecaceae identified in this study, except for *Allagoptera leucocalyx*. Fruit tree species use for charcoal production was mentioned only once, and was associated to a practice of the past, when large steamers bought wood from riparian forests dwellers to supply their boilers.

### Main uses of the wild edible plants

Locals have identified 54 species with 65 popular names. Plants names originating from Guató indigenous language, added in Table 2, which was based on Oliveira [41], were not mentioned by respondents of the Ínsua island or by respondents in other communities. The most popular names have not changed among communities. *Oryza latifolia* and *Oryza glumaepatula*, which are sympatric, were not differentiated using popular names; they were rather only identified as rice. *Hymenaea stigonocarpa* and *H. courbaril*, which are also sympatric, are used interchangeably and have also been mentioned only as jatobá, with the exception of one community (Amolar), which mentioned five popular names for both species.

Most known species were also presently used, with some exceptions, such as those used only in respondent’s childhood, such as *Victoria amazonica* and *Oryza* spp. Some uses have also been abandoned (Table 2), although the species are still used as food or are only sporadically used. Among the species mentioned as of sporadic use, *Bactris* spp. from riparian forests is emphasized, which is “consumed when someone is in the field and the hunger hits the head”; resin from fruits, such as *Cassia grandis* and *Maclura tinctoria* fruits, are abundant and extremely sweet, but their fruiting period is very short.

Fruits, seeds, leaves and flowers were mentioned for *in natura*, boiled and baked consumption, drank as fermented beverage, used for juices (using fresh or dried fruits), tea (non-medical), flavoring (used by its pleasant taste or to add to hot sugarcane syrup), and for flour, oil, vinegar, jelly and liquor production (Table 2). Fruits were the most mentioned part (44 species), followed by seeds (10), leaves (3) and flowers (1). Underground parts or aerial stems were not mentioned. *Attalea phalerata* and *Acrocomia aculeata* palms were the only mentioned leaves part, and residents said they do not use this resource anymore (Table 2).

*Acrocomia aculeata*, *Attalea phalerata* and *Vitex cymosa* were the only ones mentioned in all communities. Then, there were *Genipa americana*, *Mellicoccus lepidopetalus*, *Plinia cauliflora, Eugenia uniflora* and *Salacia elliptica*, which were mentioned by three communities. Only four species were mentioned by more than 10 % of respondents: *Hancornia speciosa, Mellicoccus lepidopetalus, Plinia cauliflora* and *Acrocomia aculeata.* Thirty species were mentioned by only one person and they had low use value.

*Acrocomia aculeata* (bocaiuva) was the species with the highest use value (Table 2), followed by *Plinia cauliflora* and *Hymenaea courbaril*. Among these, *Acrocomia aculeata* had the highest number of mentions for food uses, and only *Plinia cauliflora* was mentioned exclusively for food use. *Hymenaea courbaril* had high use value due to the large number of mentions for medicinal use, which was higher than for food. Likewise, *Pouteria glomerata*, *Bactris glaucescens* and *Copernicia alba* had a higher number of mentions for bait use in relation to food use. *Attalea phalerata* and *Copernicia alba* were the only species mentioned also for construction (leaves and stem).

Most edible uses for *Acrocomia aculeata* were mentioned by older people from different communities who reported to have used it only in the past, as for cooking oil, in the Albuquerque community, and for “chicha” (fermented or unfermented beverage), in Guató community. The oil was obtained from triturated endosperm, and then placed in boiling water. Oil use abandonment in the diet was justified due to the ease of obtaining other oils that were sold in the market, and due to the plant laborious and time-consuming process (collection and preparation time).

## Discussion

### Available vegetation types

Communities located along the Paraguay River have an important knowledge on Pantanal west edge native vegetation types wild species food use. Communities that had more available vegetation types mentioned more species, but the difference was not significant. Riparian vegetation was present in all studied communities and this vegetation type holds the greatest number of known edible species. They occur in permanent preservation areas and have its importance associated to cultivated foods production. They also act as a protection against erosion, besides containing damage to water courses, among others [42]. Thus, they contribute to aquatic habitat conservation with other food sources, especially of animal origin.

Riparian vegetation seems to be important as a diet area due to its potentially edible species richness associated with local knowledge. The fact that most species can also be used as fishing bait reflects all communities in these areas contact in relation to professional, sport or subsistence fishing activities. It contrasts with the small number of aquatic species, which originate from flooding or water courses.

Wetlands in the region have high species richness [28, 29] and offer various food resources [43]. This includes *Oryza glumaepatula* and *Oryza latifolia* extensive rice fields, which dominate the landscape during the months of April and May [44]. These species were already harvested by Guató in the past [32, 45].

The condition of a species that is ceasing to be useful can be linked to poverty, nearby resource unavailability and cultural habits loss, such as mentioned by other authors [46]. The association between wild edible plants consumption and the belief that it is “food for the poor” was also recently discussed [12,47]. According to the authors, the association with poverty (cultural acceptance) is one of the limiting factors on edible wild plants consumption.

The Guató were canoeists Indians which spent most of their time navigating, having temporary camps as their residences [32]. They were considered extinct [21] for a long time and settled on cities’ outskirts or were employed on farms. Aquatic species presently used as food lack of mentioning, especially in Guató community, may be related to this condition. Guató indigenous names absence for plants names also reflects cultural heritage loss related to the language among the descendants that currently live on Ínsua island. It also shows its little influence in other communities before being abandoned or lost, since Guató edible plants indigenous names were not mentioned by any non-indigenous respondent in other communities.

Deciduous forests have useful and well-known food species for locals. However, it was the only vegetation type related to environmental problems that threatened resources availability. A resident reported fruits scarcity in the “bushes” (deciduous forest) of his community in recent years (1990s). This would happen because of deforestation in the neighboring country, Bolivia, which would be promoting birds’ migration to this region. According to him, when trying to look for fruit in the forest, the birds would have already consumed everything. Deforestation in Bolivia was among the highest in the world in the 1990s [48] and it currently continues, especially in deciduous forests [49]. In the Brazilian Pantanal, during the study period, there were no data on deforestation in dry forests. Anyway, estimates [50] indicate that the deforested area had doubled in the 2000s in relation to the deforestation made up to 1990/91 [51]. Deforestation consequences and possible pressure and restrictions on local populations native foods access as environmental changes result need to be investigated.

Wild species that were planted in backyards by residents due to their importance as bait (*Pouteria glomerata* from the riparian forest), or as food (*Eugenia uniflora, Caryocar brasiliense* and *Hancornia speciosa* from the Cerrado), show species interest, in order to keep them closer and more available. These species cultivation techniques and handling mastering should be further investigated.

### Gender

No difference between genders was found, confirming what has been found in previous studies on wild edible plants conducted in Brazil [12,15]. Adult men in the studied communities have had enough contact with field activities, such as cattle handling, ships skipping, professional fishing and bait catching to sell to sports tourists. However, some women also hold positions that involved field activities, such as bait catching. Furthermore, in these communities, native vegetation is very close to the houses. Some information about fruit use processes, such as flours and beverages, were mentioned by older women who, due to being homemakers, are responsible for these products post-harvest processing. These communities characteristics put both men and women in a similar situation regarding the opportunity to know wild edible plants.

### Age and knowledge

Results pointed to higher knowledge among the elderly, as was also observed in other studies [3,12,14]. The lack of a younger generation (Amolar) or the fact that there were few young people (Castelo) to transfer this knowledge locally impairs knowledge transmission, as noted by [3] in a study at a community in the Argentine Patagonia. Species use abandonment, such as of *Oryza* spp., and some processing practices abandonment, such as bocaiuva and chichas making, for example, which are now known only by a few elderly people, are no longer being taken advantage of. Thus, it is also no longer known by younger generations, impacting future knowledge on local biodiversity more elaborate uses, what involves traditional technologies that were still little studied.

### Knowledge and distance from urban communities

In general, residents of the most remote communities always mentioned edible species. This suggests that this distance to an urban center promotes a closer relation with the environment, and increased wild products use in feeding. Although Albuquerque and Amolar communities total number of mentioned species is similar, the number of people who did not mention any wild edible species in Albuquerque was higher. Albuquerque community is the only one that has a small shop for manufactured food sale, and is also the most urbanized community [34]; it is the nearest to a city (Corumbá), where there is higher capability to buy food. Proximity to urban centers as an explanation for wild plants use reduction was also shown in the Bolivian Amazon [9] and in Northeastern Brazil [52]. The small number of species mentioned by Ínsua island residents was probably because most respondents had spent part of their lives in urban areas or had come to the site (Ínsua Island) recently (Table 1). This recent occupancy is because only after it was demarcated as an indigenous reserve, in 1993, that leaders encouraged Guató descendants return to the island.

### Use and use value

Fruits preference observed in this study was also observed in Argentina [10] and in the Caatinga, Brazil [52]. Fruit food use is certainly related to squash high nutritional value and also to its traditional use in the region. *Acrocomia aculeata*, for example, was the species with the highest use value and fruit number of uses, as found in a Quilombola community in Brazil [53]. It has a copper-rich mesocarp and carotenoids, and the almond has oil, protein, fiber and minerals, such as copper, phosphorus and magnesium [54].

*Acrocomia aculeata* can be found in all native vegetation types, with exception of floodplains. It has traditional uses in the region, being mentioned as the best palm used by Mbayá-Guaicurú indigenous people in the nineteenth century, whose oil was used as fuel and fruits were eaten raw, roasted or dried, as stated by [55, 56]. Mbayá-Guaicurú lived in the Paraguay River basin in the second half of the seventeenth century, and Carvalho [56] states that territory expansion occurred due to the finding of areas rich in *Acrocomia aculeata* (Namogolidi, in Guaicuru language), which ensured feeding.

It is possible that the higher use value is related to *Acrocomia aculeata* multiple use as medicine, bait and construction material. On the other hand, the small number of mentions to past uses, and only by elderly people, such as almond oil use in food or as fuel, as mentioned above, alcoholic and non-alcoholic (chichas) beverages production and flour made from the mesocarp indicate that this knowledge is in decline. This was also observed for *Attalea phalerata*, which had low use value, although having uses mentioned in the literature as a Guató food resource [20,32] and as food resource for the Midwest region [57].

Among species whose seeds are utilized, *Passiflora* spp. and *Senna occidentalis* were highlighted. Both were used for beverages production, but were also little mentioned by some locals, as palm hearts. *Senna occidentalis* has its toasted seeds used for a beverage similar to coffee [36] production, which is locally called “café de fedegoso”, replacing coffee in the morning meal.

Species with underground organs and food potential, such as *Jacaratia corumbensis*, which was mentioned in studies in Argentina [46, 58] exist in decidual forests [26], but were not mentioned by residents. No reports in the literature were found on *Jacaratia corumbensis* uses by indigenous peoples of this region, but the geographical proximity to studied areas [40, 51] suggests that peoples in the past used this resource. Nascimento et al. [13] argue that roots, tubers and seeds low consumption rate represents a waste of resources with high nutritional value that would be able to supplement the population nutritional supply, especially in drier periods of the year in the Caatinga.

### Wild edible plants and market

Abundant species with highlighted potential in the literature for economic use lack of economic utilization, such as *Hancornia speciosa* and *Caryocar brasiliense* [59,60], are related to the lack of a local or regional well-established market that stimulates this activity. It is also related to a certain distance from the communities in relation to the city, whose access is generally costly [23]. Flour made from *Acrocomia aculeata* mesocarp traded in a public market [36] and the fresh fruit sold in a free street market in Corumbá were among the few products on the local market (Corumbá).

This situation contrasts with other regions of Brazil, such as the Amazon, where wild edible plants may be found on the market since many years. Brazil nut (*Berttoletia excelsa*), for example, is today one of the main export products of the Amazon [61]. In the last decade, the Brazilian government has adopted a policy of encouraging the sustainable use of wild species. It contrasts to the previously adopted policy, especially in the 1970s, of stimulating agricultural production with deforestation for agriculture and pasture crops, and the high agribusiness incentive, on a large scale.

Plants that are marketed within communities can offer a track about resources from flora that has economic importance associated to local knowledge. These species valorization and economic exploitation can improve neglected food plants utilization and promote development (poverty reduction) and commercialization.

Wild fruits use is being stimulated in Pantanal local communities where these studies were conducted, and in other areas not included here [62]. This is aimed at highlighting wild species use in the diet, or in economic purposes, seeking also to stimulate biodiversity conservation. Among species that were the target of this study, there are *Oryza latifolia* and *Oryza glumaepatula*, which have historical value to the Pantanal and are closely related to *Oryza sativa*. They have economic exploitation potential and are abundant on the plains. This is an activity directed toward targeted species and communities in relation to one aspect, but these are certainly actions that need integrated policies to restrain communities’ extinction process and, along with it, related knowledge extinction.

As the field study in different communities was developed around 15 years ago, there were socioeconomic changes that may have influenced these communities nowadays. It is possible to highlight, for example, one project dealing with wild food plants developed in some of these communities, aiming to improve their incomes [62]. In another example, in the Amolar community, most of the interview elderly people died, or their families do not live there anymore. The two situations are antagonistic, because the first stimulates local knowledge and use (with wild plant food use in the diet and marketing) and the second involves elderly rural population reduction, which held more knowledge that could be shared with future generations.

## Conclusions

As expected, people from communities more distant from urban area and the elderly, both men and women; hold more knowledge on wild edible species that can still be accessed in the communities along the Paraguay River.

The greater availability of vegetation types does not influence the richness of plants known by the communities. These aspects need further study taking into account the total number of species in each physiognomy.

The wild edible species have been little used, as evidenced by the low rates of use value and species used in the past as well as important uses of the past are no longer used, suggesting an abandonment or loss of knowledge of wild edible plants of the Pantanal by the communities studied.
